# Photosensitized production of functionalized and unsaturated organic compounds at the air-sea interface

**DOI:** 10.1038/srep12741

**Published:** 2015-08-05

**Authors:** Raluca Ciuraru, Ludovic Fine, Manuela van Pinxteren, Barbara D’Anna, Hartmut Herrmann, Christian George

**Affiliations:** 1 Université de Lyon 1, Lyon, F-69626, France; CNRS, UMR5256, IRCELYON, Institut de Recherches sur la Catalyse et l’Environnement de Lyon, Villeurbanne, F-69626, France; 2 Leibniz-Institut für Troposphärenforschung e.V. (TROPOS), Atmospheric Chemistry Dept. (ACD), Permoserstraße 15, 04318 Leipzig, Germany

## Abstract

The sea-surface microlayer (SML) has different physical, chemical and biological properties compared to the subsurface water, with an enrichment of organic matter i.e., dissolved organic matter including UV absorbing humic substances, fatty acids and many others. Here we present experimental evidence that dissolved organic matter, such as humic acids, when exposed to sunlight, can photosensitize the chemical conversion of linear saturated fatty acids at the air-water interface into unsaturated functionalized gas phase products (i.e. saturated and unsaturated aldehydes and acids, alkenes and dienes,…) which are known precursors of secondary organic aerosols. These functionalized molecules have previously been thought to be of biological origin, but here we demonstrate that abiotic interfacial photochemistry has the potential to produce such molecules. As the ocean is widely covered by the SML, this new understanding will impact on our ability to describe atmospheric chemistry in the marine environment.

The air-water interface of the oceans is of major environmental importance as its chemical composition, driven by chemical and physical processes in the ocean, does not only influence the organic fraction of marine aerosol produced by sea spray processes^1^,^2^ but also controls trace gas deposition to the ocean and may be involved in secondary organic aerosol formation in the marine boundary layer. These aspects are central to atmospheric composition changes, air quality and associated climate change. Hence, a better chemical characterization of the oceanic surface microlayer (SML) and its chemical processing is highly desirable to understand the variable biogeochemical and physical controlling factors allowing assessment of the contributions and consequences of global environmental change^3^. The SML, covering up to 70% of the Earth’s surface^4^, has an enrichment of organic matter i.e., dissolved organic matter including UV absorbing humic substances, fatty acids, amino acids, proteins, lipids, phenolic compounds, as well as trace metals, particulate matter and microorganisms^3^. Here we show that under solar illumination, compounds present in the SML release highly reactive unsaturated organic compounds to the atmosphere from the water surface, via a photosensitized process.

There is a growing body of evidence that the heterogeneous reactive loss of gas phase NO_2_ and ozone at surfaces containing photoactive compounds may be significantly enhanced under illumination^5^,^6^. In this context, Reeser *et al.*^7^ studied the heterogeneous reaction between gas-phase ozone and chlorophyll present at the air-salt water interface and showed that the kinetic mechanism is altered in the presence of actinic radiation, throughout the entire near-UV and visible spectrum. Similar observations were made by Jammoul *et al.*^8^ with simple aromatic ketones, present at the air sea interface, instead of chlorophyll. Otherwise, to date, there has been little or no attention paid to atmospherically relevant photochemistry taking place at the oceanic air-water interface^3^. It is speculated that some photochemical processes could be enhanced, but there is little experimental evidence of this up to now^9^,^10^.

Air-water interfaces are widespread in the environment. Interestingly, peptide bond formation at the air-water interface has been recently observed using water-soluble amino acid esters through Cu^2+^ coordination. The formation of peptide bonds in the presence of bulk water is thermodynamically disfavoured as these bonds require the elimination of a water molecule for each peptide bond formed. However, the hydrophobic environment of the air-water interface has been shown to favour peptide formation^11^. This highlights the fact that organic enriched interfaces exhibit chemistry strongly different compared to homogeneous bulk media.

In the present study, we explored some atmospherically important consequences of photochemistry at the sea surface microlayer. The experiments were designed in such a way that thin (up to a monolayer) organic layers on bulk water were exposed to light, mimicking solar irradiation, whereas the underlying bulk water contained dissolved organic matter in the form of humic acids, well known natural photosensitizers^12^. Photochemistry in the surface ocean is dominated by dissolved organic material (DOM), among which humic substances do correspond to an important family^13^. When exposed to light, the chromophores contained in the humic acids will be excited. Subsequently, they initiate the degradation of the organic surfactant at the air-water interface from their triplet state. Such processes in the bulk water are well documented^13^ and are known to initiate a series of potential oxidative pathways such H abstraction, charge transfer reactions, or the production of hydrated electrons, HO_x_ or singlet oxygen, all of which have the potential to chemically degrade aqueous organic compounds, leading to the formation of alcohols, aldehydes or ketones the structure of which will depend on their respective precursor molecules. Therefore, under illumination, DOM will initiate the photo-degradation of the organic surfactants at the interface. The gas phase products arising from the photoinduced reactions were analysed by means of high resolution proton transfer mass spectrometry (PTR-ToF-MS), using different ionization agents, in order to exclude any possible interference in the mass attribution.

## Results

Authentic SML samples from Norway were used in this study. These samples were collected by means of the glass plate technique, off the coast of the Raune fjord (Bergen, Norway) and are therefore representative of “coastal samples”, which can be more continentally influenced compared to samples collected in the open ocean. Sampling was performed on a sunny day under calm conditions (low wind speed of 4 m s^−1^) at 1 pm UTC (Coordinated Universal Time) and the collected SML film was around 200 μm thick. Salinity as well as dissolved organic carbon (DOC) and particulate organic carbon (POC) content of the SML sample are listed in Table 1. The high POC concentrations in the sample are probably due to bloom conditions and chlorophyll-a was relatively high, with a concentration value of 2 μg L^−1^ during the sampling period.

Dissolved organic matter (DOM) concentrations in surface water typically range from 0.1 mg L^−1^ to 20 mg L^−1^ and is mainly composed of humic substances; the sea surface microlayer contains up to ten times the concentration of DOM compared to its bulk water concentration^13^. In the SML samples, 30 mgL^−1^ humic acid was added to mimic the presence of the DOM in underlying subsurface water. The main difference in chemical composition between terrestrial and marine DOM is the level of H/C, N/C and O/C and it has been shown that the ratios for the humic acids used in our study are ranging within the values reported for marine humic acids^14^. These samples filled ca. half of a small Quartz cell (2 cm diameter and 5 cm length) which was irradiated by means of a Xenon lamp and continuously flushed with purified air. Background experiments were performed on an empty reactor, on deionized water and on solutions containing only the humic acids. In all background experiments no gas phase products were observed in the different ionization modes of the PTR-ToF-MS (H_3_O^+^ or NO^+^).

**Figure 1:**
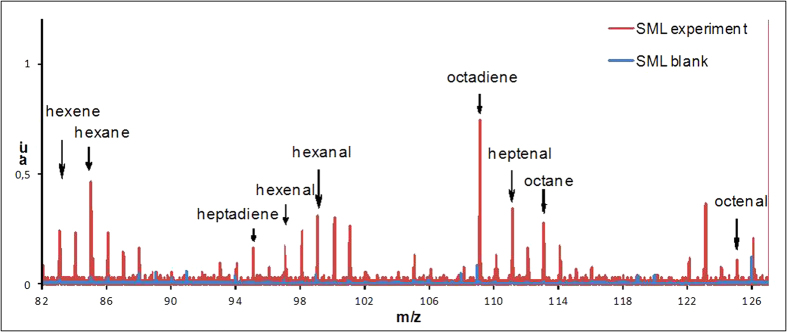
Typical irradiation spectra on authentic samples. Blue spectra: SML blank, red spectra: SML containing humic acid (30 mg L^−^1). The figure shows the formation of a certain number of compounds measured by PTR-ToF-MS in NO^+^ ionization mode (see also Table S1)

When irradiating the SML samples, the expected photosensitized degradation of the SML led to the production of a large variety of oxidized products (Fig. 1 and Table S1) such as saturated aldehydes, ketones, etc. However, in addition, these experiments also revealed the production of a series of unexpected, unsaturated and functionalized products (i.e. hexene, hexenal, heptadiene, octadiene) as exemplified in Fig. 1 (see also Table S1). The importance of such observations is that the mentioned molecules are reactive, for instance with ozone, and could therefore initiate new chemical transformations in the gas phase close to the air-water interface. This would represent a new chemical photosensitized source of precursors for radicals and aerosols, while our current understanding points only to biological sources for such unsaturated compounds.

To gain more insights into the underlying chemistry that leads to the production of these unexpected compounds, further experiments were performed on synthetic SML samples. These experiments were performed using organic layers of a known surfactant, nonanoic acid (NA), on a salt solution with humic acids added again as an environmentally relevant photosensitizer. This allowed us to elaborate mechanistic information under controlled (and clean) conditions and to extend this knowledge to the (less controlled) real environmental conditions.

Fatty acids are ubiquitous in the environment; nevertheless, to our best knowledge, the nonanoic acid concentration in SML is unknown. This fatty acid is a known product from the oxidation of oleic acid; elevated concentrations of C9 acid have been attributed to the ozonolysis of oleic acid, the latter known to be enriched in the surface microlayer of the ocean^15^. King *et al.*^16^ investigated the reaction of ozone with oleic acid monolayers on water and reported a high yield of nonanoic acid (87%) which remained at the surface of the water. Furthermore, particle and vapor-phase organic compounds measurements showed 4.9 ng m^−3^ nonanoic acid in fine particulate organic acids in marine areas^17^,^18^. Here we use nonanoic acid as a simple proxy for fatty acids.

After mixing humic acid (photosensitizer) and nonanoic acid (surfactant) in water, upon illumination, a large number of oxidized gaseous products, as listed in Table S1 and shown in Figure S1 were observed. These compounds are produced upon the oxidation of nonanoic acid, initiated most probably by a H-abstraction from the alpha position to the carboxylic function, leading to the breakdown of the acid. When exposed to light, it is known that excited triplet state^12^ chromophores in humic acids can initiate degradation of organics, presumably including the organic surfactant at the air-water interface, via a series of oxidative pathways such H-abstraction, charge transfer reactions, hydrated electrons, HO_x_ or singlet oxygen production, leading to the formation of alcohols, aldehydes or ketones which structure depends on their parents molecule. The same pattern of products was observed in water and salt containing solutions (prepared as a proxy for seawater, Table S1).

**Figure 2:**
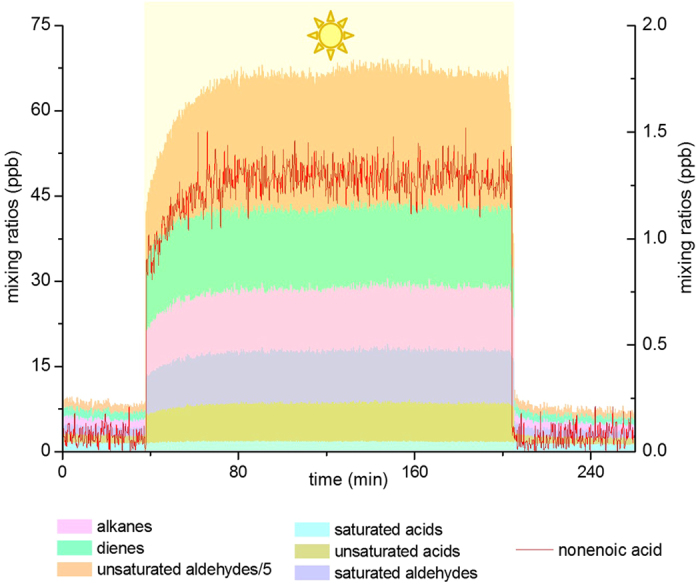
Typical irradiation experiment of a solution containing salt water, humic acid (30 mg L^−1^) and nonanoic acid (1 mM) showing the formation of different classes of organic compounds (sum of saturated and unsaturated acids, saturated and unsaturated aldehydes, alkanes and dienes), nonenoic acid (red line) measured by PTR-ToF-MS with H_3_O^+^ as reagent ion. The right ordinate axis represents the mixing ratios of nonenoic acid, while the left is representing the mixing ratio of the other classes of compounds.

Surprisingly, in addition to these expected products, and similarly to the experiments performed on authentic samples, the photoinduced formation of alkenes, dienes, and series of C2-C9 unsaturated aldehydes was also observed. These compounds were detected in the PTR-ToF-MS spectra at m/z 57, 85, 99, 113, 127 and 141 which are indicative of the compounds propenal, pentenal, hexenal, heptenal, octenal and nonenal, respectively. These chemical attributions were verified by other, independent means (i.e., Automatic Thermal Desorption Gas Chromatograph Mass Spectroscopy (ATD-GC-MS), PTR-ToF-MS using NO^+^ ionization mode) in order to avoid any misattribution. The formation of such unsaturated compounds in an oxidative medium is unfavorable in bulk aqueous environments^19^ as double-bond formation could occur by water elimination. But as shown in Fig. 2, the more hydrophobic organic enriched environment of the air–water interface appears to establish a favorable venue for the production of unsaturated gaseous compounds. Figure 2 shows the outcome of a typical experiment, where the above mentioned series of unsaturated compounds are readily produced after switching on the light. The timescale required to produce these chemical products must be very short as the residence time of the carrier gas in the quartz reactor is in the order of a few seconds, depending on the actual gas flow. One of the first possible unsaturated products would correspond to a C9 molecule such as nonenoic acid, whose formation as a function of irradiation time is also shown in Fig. 2. It can be seen that a steady state concentration is reached very rapidly and maintained through-out the irradiation period. We stress here that none of these products were observed using only water, water with humic acid alone or water with the surfactant alone (Figures S2 and S3).

**Figure 3:**
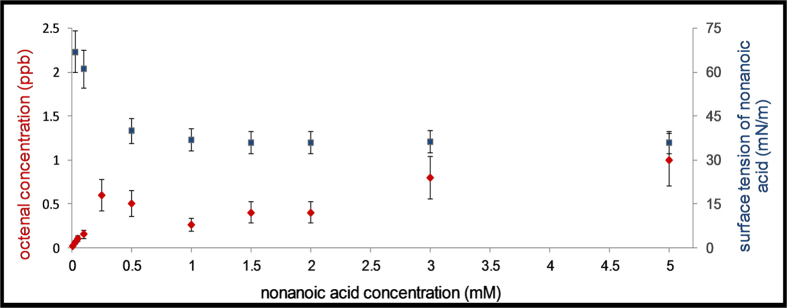
Evolution of octenal concentration (left ordinate axis) and of the surface tension (right ordinate axis) as a function of bulk nonanoic acid concentration. The errors in octenal concentration are estimated at 30% and for the measured surface tension at 10%.

Due to their molecular structure, surfactants are enriched at the air/water interface, with their surface excess concentration related to surface tension^20^. If a surfactant is important to the reaction the chemical reaction rate and its product yields will not follow bulk phase concentrations but rather will track those of the surface component. This, in turn, is related to the surface tension, via the Gibbs equation^20^. With increasing bulk concentration of nonanoic acid, the surface tension will decrease and reach almost a steady state when the surface is fully covered by the surfactant (see Fig. 3). This surface tension decrease indicates an increase in the surface concentration of NA, finally reaching an asymptotic value, despite continuously and linearly growing bulk concentrations. The implication of this behaviour on product yields is that they should increase linearly with surfactant concentration for bulk processes but reach an asymptotic value for interfacial processes. The behaviour observed here is depicted in Fig. 3 for octenal, one of the main products identified. The gaseous concentration levels off at around 1 mM of NA i.e., tracking the behaviour of surface tension. This clearly points to an interfacial process involving the surface concentration of NA, in which only monolayers of organic compounds trigger the chemistry, without the requirement of large concentrations of fatty acids in the bulk solution.

**Figure 4:**
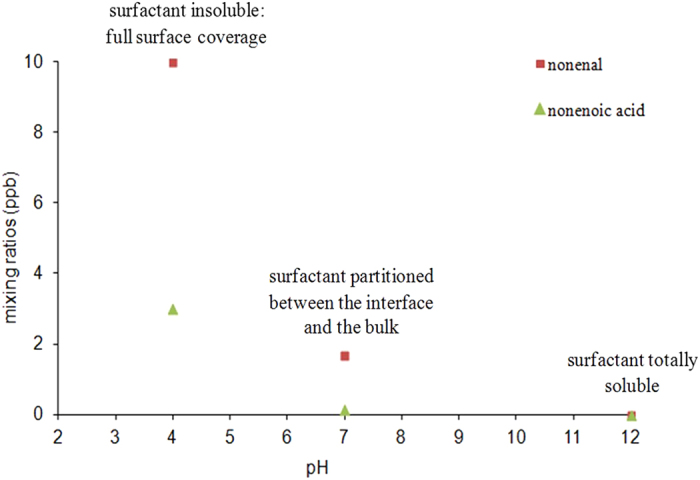
Evolution of gaseous nonenal and nonenoic acid concentration as a function of the pH of the solution.

Experiments as a function of pH have also been performed using nonanoic acid (pKa = 4.9) as surfactant, within a pH range of 4 to 12. After increasing the pH of the solution to 12, none of the unsaturated compounds were identified in the PTR-ToF-MS spectra. Figure 4 illustrates the mixing ratios of nonenal and nonenoic acid as a function of the pH of the solution. This can be explained by an increase of the solubility of the acidic surfactant under alkaline conditions, due to its acid-base dissociation, leading to a strongly reduced surface concentration. These observations support the hypothesis that the organic surfactant must be present at an interfacial layer rather than the bulk liquid; as with a reduced surface concentration no chemical reactions could be observed.

## Discussion

The core of the experiments presented above have been performed on synthetic samples, using nonanoic acid as a proxy for fatty acids, and are showing that interfacial photochemistry has the potential to produce unsaturated compounds as identified and monitored mainly by means of a PTR-ToF-MS. While the approach represents a simple proxy for oceanic surface water, a smaller number of experiments has been performed on authentic SML samples, highlighting that these observations can be extended also to real marine conditions, bearing in mind, nevertheless, that this extrapolation currently is based on a limited number of samples. Our observations show that the observed chemistry lead to the emission of a variety of reactive compounds i.e., aldehydes, mono and poly-unsaturated VOCs all of which may be photolyzed or react with ozone, triggering atmospheric reactions which will lead to low volatility products and thus aerosol formation^21^. The observed photochemistry depends upon the ability of the oceanic SML to promote close contact and increased concentration among reagents compared to the bulk, thus enabling the formation of products at the interface which may easily be released to the gas phase. While most of these compounds were known to have some biological sources, the present study reveals that interfacial photochemistry may form an abiotic source of the unsaturated and functionalized chemical species discussed here.

**Figure 5:**
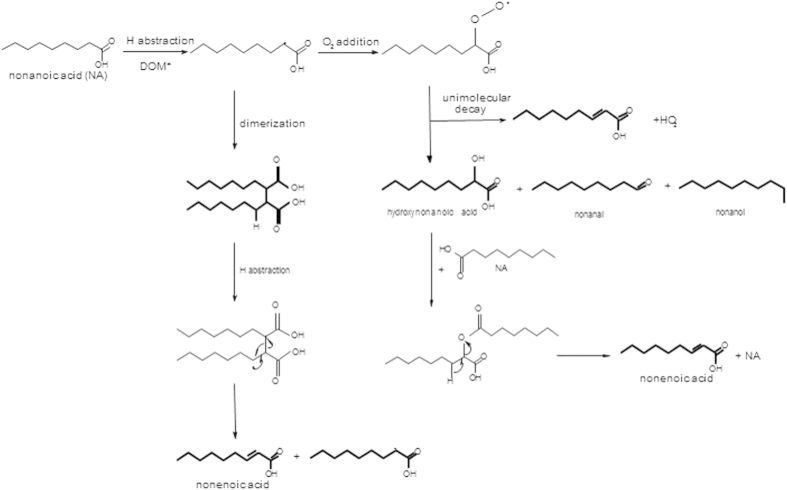
Suggested reaction mechanism leading to the chemical production of unsaturated compounds at the air-sea interface; products in bold have been observed in this study; identified products are in bold in this figure.

To explain speculatively (see Fig. 5) how nonanoic acid might be converted, at the air-water interface, into the gaseous compounds listed above, one first needs to identify the initial oxidant triggering the chemistry. Humic acids, and more specifically aromatic ketones, are known to yield reactive species in aqueous solutions, i.e. HO_x_ or O_2_^–^ or they can sensitize the formation of singlet oxygen^22^,^23^. As soon as these species are formed, they react rapidly with a number of organic compounds. Excited humic acids can initiate chemical reactions via multiple pathways i.e., charge exchange reactions or H-abstraction. In the case of a saturated carboxylic acid, both are possible. The observation demonstrates the capability of excited humic acid to react with nonanoic acid. In the case of H abstraction, the initial step would then be the reaction of nonanoic acid with OH radicals or by direct abstraction of an H-atom at the alkyl chain by some excited humic acid moiety, to form H_2_O and a carboxyalkyl radical (which could not be measured here). The addition of oxygen to this radical could then lead to α-peroxyl radical, the chemistry of which has been reviewed by Von Sonntag and C Schuchmann^24^ where the monomolecular degradation (intramolecular arrangement) could lead to nonenoic acid production, which was identified in this study, while the recombination pathway, possibly favoured at the interface due to the concentrating effect, would lead to the other α-oxygenated products observed by means of a PTR-ToF-MS, such as hydroxynonanoic acid or α-keto acids which have also been shown to be photochemically active (in the case of pyruvic acid^25^). Nonenoic acid could then be formed via acid catalyzed dehydration of the hydroxynonanoic acid as hydrogen ions are available due to the acidity of the solution. Two carboxyalkyl radicals can also recombine to form a dimer which may also suffer H-abstraction, leading to nonenoic acid and to new radical intermediates. Analogous dimerization reactions have been observed in the literature in the case of oxooctanoic acid^26^. While being still speculative, Fig. 5 nevertheless explains the formation of the observed unsaturated gas phase products. For completeness, several other classical oxidation steps needs to be added to explain the observation of shorter oxidized products (produced upon decarboxylation of the parent molecule).

A number of peroxyl radicals can eliminate HO_2_, but they require a substituent which acts as charge stabilizer. Amino groups or double substituted alkoxy peroxy intermediates have been shown to greatly accelerate this elimination process^24^. Unimolecular decay of a peroxyl carboxylic acid has not yet been reported, but the carboxylic function might trigger this pathway. Bimolecular decay of peroxyl radicals derived from carboxylic acids has already been experimentally investigated on aqueous interfaces. Enami *et al.*^27^ showed the formation of peroxyl radicals at the water interface region; reacted within 50 μs to produce alcohols and carbonyls. Our findings are consistent with their work.

In addition to reactions described above, it has been shown that disproportionation reactions of peroxy radicals can lead to the formation of alcohols and aldehydes, as depicted in PTR-ToF-MS spectra, these species leading to the formation of extremely low volatility organic compounds (ELVOC)^28^,^29^.

Recently, there has been renewed interest and debate about the propensity of ions at interfaces^30^. Investigations of ions at the surface versus bulk showed an enhancement of hydronium ions at the topmost layer of water and aqueous acid solution, making the air-water interface acidic^31^; although other experiments showed enhanced surface concentration of hydroxide and depleted hydronium due to accumulation of hydroxide anions at the air-water or oil-water interfaces^32^. Direct experimental results and calculation reports showed that hydronium ions are present at a higher concentration at the surface than hydroxide ions, yielding an acidic pH (e.g. carboxylic acids tend to deprotonate at the water surface^33^). The surface may then be rich in hydronium ions which would thus be available to catalyze reactions there^27^,^33^.

A key feature of reactions which are constrained to occur at an interface is that they may favour protonation and self-reaction (due to higher concentrations) which will lead to the formation of dimers (Fig. 5) as suggested by Griffiths *et al.*^26^. Following dimer formation, any subsequent hydrogen abstraction reaction will ultimately result in shorter alcohols, acids, aldehydes or ketones which can in turn lead to the formation of various unsaturated products as identified in the PTR-ToF-MS spectra (Table S1).

Finally, to ensure the applicability of reaction Fig. 5, future work shall focus of a full assessment of all products in all phases (liquid and gaseous, not excluding the interface), on the importance of the chemical functionalities of the initial surfactant molecule, and to get closer to environmental conditions the use of larger number of authentic samples. The latter may then be useful to reveal the seasonality and geographical importance of the interfacial abiotic production of VOCs and OVOCs.

The largest environmental interface is certainly the one formed by the oceans. Wurl *et al.*^34^ studied the surfactants concentration at the air/sea interface in different regions of the ocean (subtropical, temperate, and polar). These authors concluded that the ocean surface microlayer is enriched with surfactants to a much larger extent than previously recognized, especially in the Norwegian Sea in summer and fall. According to their data, during these periods, it can be assumed that the sea is fully covered by an organic layer as thin as a monolayer. Accordingly, the chemistry exemplified in Fig. 5 for one given simple fatty acid may be a wide spread source of functionalized VOCs. A measurement campaign at a coastal site observed the presence of insoluble organic films coating the particles in marine air leading to water uptake suppression. The authors speculated that the coating might be explained by vegetation sources, although sea-surface microlayer may also represent an important source of secondary film-forming organic compounds^35^. The results from the present study could explain their observations, showing that the particles’ precursors could be of marine origin without any biological activities. Furthermore, unsaturated aldehydes and mainly polyunsaturated ones have been shown to be widely spread in oceanic surface water (in this case referring to depth up to 3 meters) and produced from diatoms. Total polyunsaturated concentrations ranged from 0 to 4.18 pmol per cells in 1 L, the most abundant molecules being heptadienal, octadienal and decadienal^36^. Such concentrations and the similarity of the molecules measured in ocean surface waters and in the present study is intriguing and certainly tends to show that potentially non-biological sources have to be considered: photochemistry at the air-sea interface could also be significant.

Through these results, we have demonstrated formation of functionalized and unsaturated compounds at the air–water interface arising from the photosensitized degradation of organic surfactants, while previous knowledge points only to biological sources for such molecules. The interface is a specific venue for such reactions and we suggest that it promotes self-reactions of peroxyl radical intermediates differently than dilute bulk solutions, while the photosensitizer facilitates the production of oxidative centers (radicals or triplet states). However, surfactant covered (even if not fully covered) water–air interfaces are widespread and typical for the surface of oceans, lakes, and atmospheric aerosols, providing an auspicious environment for this chemistry which produces reactive unsaturated compounds that in turn may affect a variety of atmospheric processes, such as particle production.

## Methods

**Figure 6:**
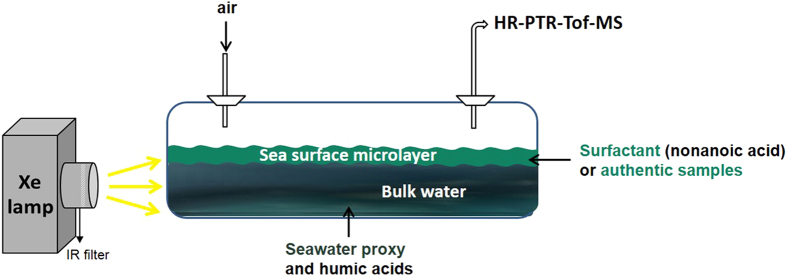
Schematic of the experimental layout.

The setup used to study the photochemical reactions at the water surface consists of a Quartz cell (2 cm diameter and 5 cm length) filled with 7 ml of synthetic mixtures of sea surface microlayer (SML) or natural SML samples (Fig. 6). The artificial SML mixtures contained an aqueous solution of NaCl, NaBr, NaI, a photosensitizer (humic acids) and an organic surfactant (e.g., hexanol, octanol, nonanoic acid). The solutions have been irradiated by means of a Xenon lamp (spectral profile is given in Figure S5 and compared to the actinic flux values at the Earth’s surface) and a 200 sccm of purified air was flowing through the quartz cell. The photodegradation of the artificial SML was carried out at various concentrations of nonanoic acid ranging from 0.25 and 5 mmol L^−1^. The gas-phase products were investigated by proton transfer reaction mass spectrometry (PTR-ToF-MS) with H_3_O^+^ and NO^+^ as reagent ion. Blanks experiments were routinely performed on 18 MΩ deionized water and solutions containing the surfactant or the humic acids, to assess background signals when irradiating.

For the experiments with artificial SML, a salt solution composed of 1 mol L^−1^ sodium chloride (99+%, Sigma-Aldrich), 1 mmol L^−1^ sodium bromide (99+%, Sigma-Aldrich), 10^−3^ mmol L^−1^ sodium iodide (99+%, Sigma-Aldrich) with additional concentrations of humic acids (Fluka) was prepared by dissolving known amounts of the products in 18 MΩ deionized water. The surfactant monolayer was prepared by adding a known concentration of nonanoic acid (97%, Sigma-Aldrich) in these solutions. The pH of the solution was raised to approximately 8 using a sodium hydroxide solution; experiments were performed using solutions to which base was not added, these having a pH in the range of 4–5.

When using the SML samples, 30 mgL^−1^ humic acid were added directly into the SML to mimic the presence of the DOM in underlying subsurface water. In this case, no artificial salt water was added into the cell.

In some experiments, nonanoic acid was previously purified by bubbling ozone before irradiation. In this case, the PTR-ToF-MS spectra showed no differences compared to those acquired without purification. All the experiments presented in the paper represent the results without purification.

Measurements were carried out using a commercial SRI-PTR-ToF-MS 8000 (Selective Reagent Ionization Proton Transfer Reaction Time of Flight Mass Spectrometer) instrument from Ionicon Analytik GmbH (Innsbruck, Austria) in its standard configuration (V mode)^37^. The PTR-ToF-MS sampled continuously 50 sccm from the emissions stream through 1.5 m of 6 mm i.d. peek tubing. No particle filter was placed at the inlet to avoid artefact from possible organics deposition on the filter. Internal calibration of the TOF data and peak extraction were performed according to the procedure described in detail by W. Lindinger *et al.*^37^. PTR-ToF-MS spectra were collected at a time resolution of 8s. Measurements using H_3_O^+^ ionization were performed using a drift voltage of 600 V, drift temperature of 60 °C and a drift pressure of 2.25 mbar resulting in an E/N of about 130 Td (1Td =10^−17^ cm^2^V^−1^). The ionization conditions in the NO^+^ mode were maintained at a drift voltage of 600 V and a drift pressure of 2.25 mbar. The instrument was operated at an E/N value of 132 Td. The volatile organic compounds concentrations are expressed in ppbv and they have been calculated according to the formula described in Cappellin *et al.*^38^. We have used the same value of *k* = 2 × 10^−9^ cm^3^/s for all masses excepting isoprene, which has a value of *k* = 1.94 × 10^−9^ cm^3^/s^39^. Uncertainties in our data may arise from systematic errors in the concentration determination since the accuracy for compounds concentration has been estimated using calculated values for the collision rate constant which should equal the reaction rate constant within ±30%^40^. Typical detection limits, precisions and accuracies for the selected VOCs are 5 ppt, 3% and 6%, respectively.

One of the main weaknesses of PTR-ToF-MS is that the VOC identification relies solely on mass spectrometry; therefore comparison of PTR-ToF-MS measurements to those by means of a GC-MS technique was used for accurate identification of individual VOCs. Analysis of VOCs were then performed by Automatic Thermal Desorption (ATD Markes Thermal Desorber Unity with an Markes Autosampler Ultra 2) coupled with GC/MS, using an Agilent 6890 GC (DB-VRX column of 60 m × 0.25 mm × 1.4 μm, J&W, 122–1564) and an Agilent MSD 5973 N MS with electron impact ionization mode. A Tenax sorption tube of 200 mg (L = 3 1/2”, OD = 1/4”) has been used (ATD-GC/MS conditions are detailed in Table 2).

The surface tension measurements have been performed using a Kruss tensiometer K6 (measuring range between 0 and 90 mN/m).

The glass plate technique was used to sample the sea surface microlayer. A glass plate with the dimensions of 500 × 250 × 4 mm was vertically immerged in the water, slowly removed and the surface film was wiped off with a Teflon wiper. After sampling, the samples were immediately frozen and stored at −20 °C until analysis. No biocide was added. Sub-surface water was collected in order to determine enrichment factors of organic carbon. Enrichment factor (EF) was defined via the concentration of organic carbon in the SML and the concentration in the sub-surface water. For sub-surface water sampling a glass bottle was mounted on a telescopic rod and opened in a depth of 2 meters. The authentic sea surface microlayer samples were analyzed for salinity, dissolved organic carbon (DOC) and particulate organic carbon (POC) and the results are showed in Table 1.

Dissolved organic carbon was determined with a total organic carbon analyzer (Shimadzu, Kyoto, Japan). The oxidation of the carbon within the sample was performed at pH 2 by adding 300 μL of the solutions (SML/sub-surface water, filtered over a 0.2 μm syringe filter) to a catalyst (aluminium oxide/platinum) held at a temperature of 720 °C. The formed carbon dioxide was detected by non-dispersive infrared (NDIR). Salinity was determined with a refractometer (RHS-10 ATC). The detection limit for POC was 1.8 μmol/L for a filtration volume of 1000 mL. For particulate organic carbon determination the SML sample was filtered onto pre-combusted (8 h at 450 °C) glass fibre filters (GF/F, Whatman) and measured with a Sunset Laboratory Dual-Optical Carbonaceous Analyzer (Sunset Laboratory Inc. U.S.A.). Temperature control was after the EUSAAR-2 protocol: www.atmos-meas-tech.net/3/79/2010/. All data were corrected for blank values.

## Additional Information

**How to cite this article**: Ciuraru, R. *et al.* Photosensitized production of functionalized and unsaturated organic compounds at the air-sea interface. *Sci. Rep.* **5**, 12741; doi: 10.1038/srep12741 (2015).

## Supplementary Material

Supplementary Information

## Figures and Tables

**Table 1:** SML sample characteristics.

**Sampling date**	**10.05.2011**
Salinity (Practical Salinity Units)	31
DOC SML (μmol L^−1^)	148
POC SML (μmol L^−1^)	87
EF (DOC)	1.6
EF (POC)	2
DOC subsurface water (μmol L^−1^)	94
POC subsurface water (μmol L^−1^)	44

**Table 2:**
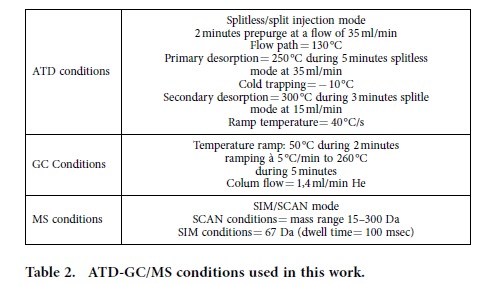
ATD-GC/MS conditions used in this work.
