# Supplementary material for: Photosensitized production of functionalized and unsaturated organic compounds at the air-sea interface
Source: Sci Rep. 2015 Aug 5;5:12741. doi: 10.1038/srep12741 (PMC4650702; doi:10.1038/srep12741)
Supplement: Supplementary Information [file srep12741-s1.pdf]

Photosensitized production of functionalized and unsaturated organic compounds at the air-sea interface

Supplementary information

Raluca Ciuraru[1], Ludovic Fine[1], Manuela van Pinxteren[2], Barbara D'Anna[1], Hartmut Herrmann[2], Christian George[1]*

[1]*Université de Lyon 1, Lyon, F-69626, France; CNRS, UMR5256, IRCELYON, Institut de Recherches sur la Catalyse et l'Environnement de Lyon, Villeurbanne, F-69626, France*

[2]*Leibniz-Institut für Troposphärenforschung e.V. (TROPOS), Atmospheric Chemistry Dept., Permoserstraße 15, 04318 Leipzig, Germany*

* To whom correspondence should be addressed. E-mail: christian.george@ircelyon.univ-lyon1.fr

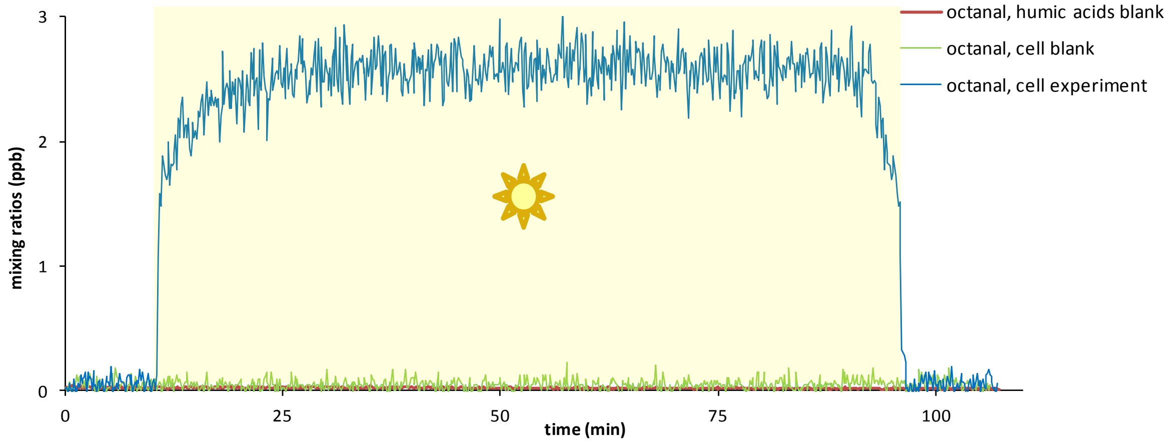

Figure S1. Typical irradiation experiments on different samples. Red line: humic acids blank, green line: quartz cell blank and blue line: a solution containing salt water, humic acid (30 mg L^{-1}) and nonanoic acid (1mM). The figure shows the formation of octanal measured by PTR-ToF-MS, this compound being an expected oxidation product arising from photodegradation of fatty acids.

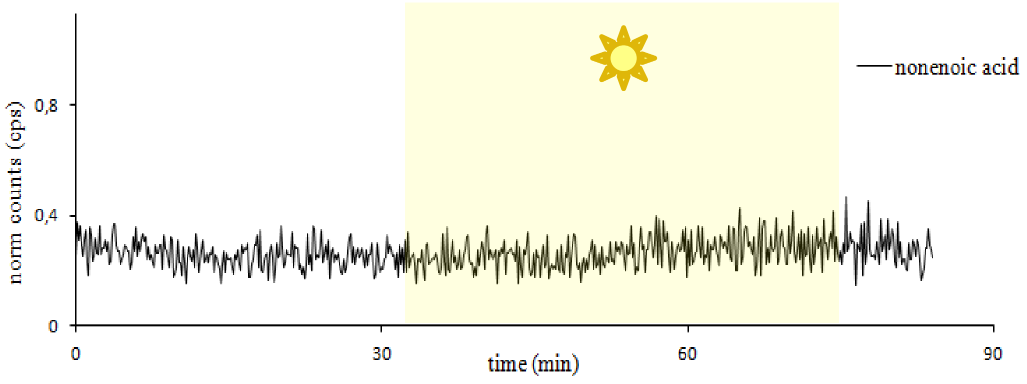

Figure S2. Nonenoic acid signal in NO^+ ionization mode during an irradiation experiment of a solution containing water and nonanoic acid

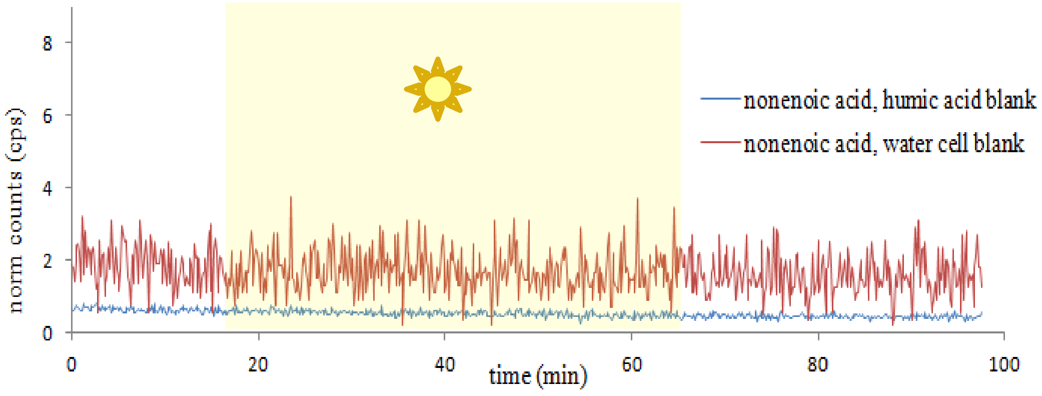

Figure S3. Nonenoic acid signal in H_3O^+ ionization mode during an irradiation experiment of a solution containing either water or humic acid

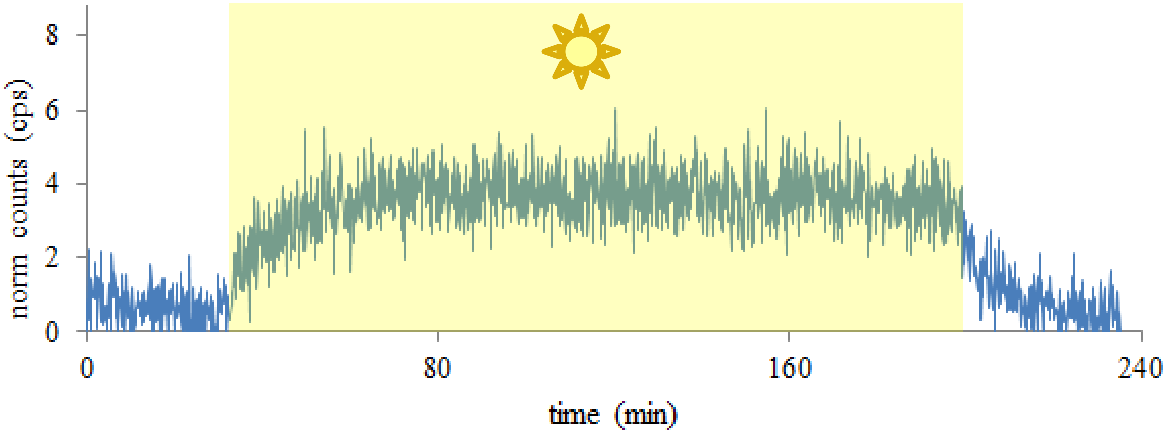

Figure S4. Nonenoic acid signal on a typical irradiation experiment: a solution containing salt water, humic acid (30 mg L^{-1}) and nonanoic acid (1mM)

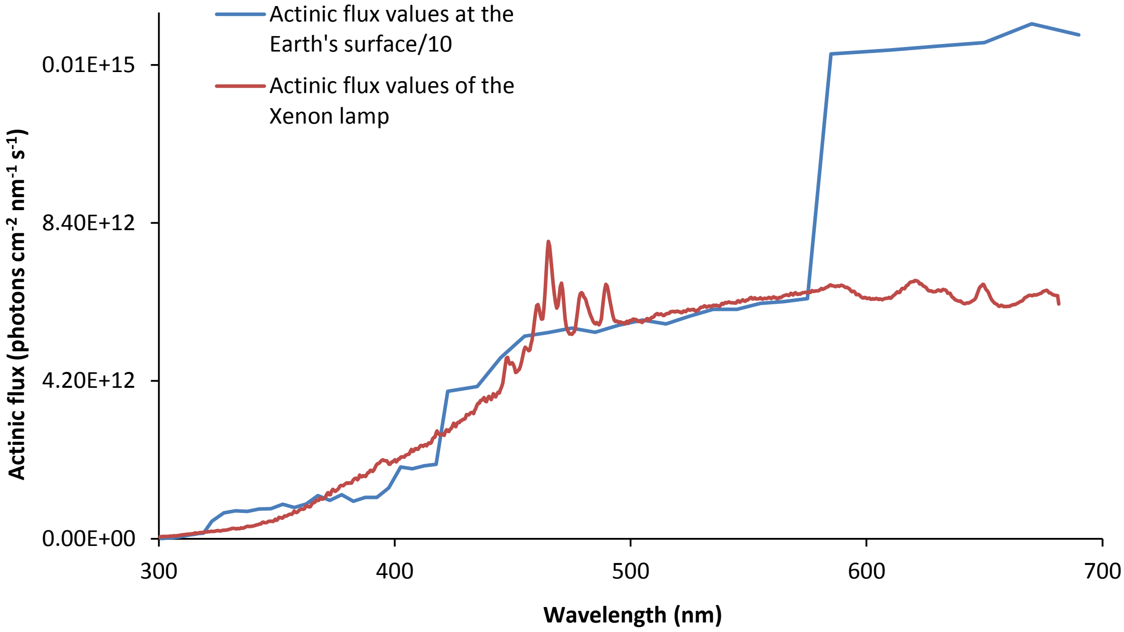

Fig S5. Spectral profile of the 150 W Xenon lamp used for irradiation filtered by a water film to remove infrared radiation (red line) compared to the actinic flux values at the Earth's surface taken from[1]

Table S1. Identified compounds by PTR-ToF-MS and ATD-GC/MS for artificial and authentic SML samples

Class	Compound	Nonanoic acid		Authentic SML samples** (ppbv)
		PTR-ToF-MS Mixing ratios (ppbv)*	ATD-GC/MS	
Saturated aldehydes	acetaldehyde	3.3	x	0.4
	propanal	2	x	0.4
	butanal	0.1	x	0.1
	pentanal	0.3	x	0.1
	hexanal	0.2	x	0.05
	heptanal	0.1	x	0.04
	octanal	2.4	x	0.1
	nonanal	0.15	x	-
	decanal	0.15	x	0.01
Unsaturated aldehydes	butenal	0.3		-
	methacrolein	x	x	0.1
	pentenal	0.6	x	0.05
	hexenal	0.15		0.02
	heptenal	0.1		0.03
	octenal	0.4		-
	nonenal	15		-
Ketones	methyl ethyl ketone	x	x	-
	methyl vinyl ketone	x	x	0.008
	pentanone	x	x	0.009
	pentenone	x	x	0.02
	hexanone	x	x	0.009
	hexenone	x	x	0.03
	heptanone	x	x	0.005
	octanone	x	x	0.009
	octenone	x	x	0.008
Alcohols	pentanol	8		-
	hexanol	0.2	x	-
	heptanol	0.6		-
	octanol	0.3	x	-
	nonanol	0.5		-
Saturated acids	pentanoic acid	0.02		-
	hexanoic acid	0.04		-
	heptanoic acid	0.08		-
	octanoic acid	0.08		0.005
	nonanoic acid	x	x	-

Class	Compound	Nonanoic acid		Authentic SML samples** (ppbv)
		PTR-ToF-MS Mixing ratios (ppbv)*	ATD-GC/MS	
Unsaturated acids	propenoic acid	x	x	-
	butenoic acid	0.2	x	-
	pentenoic acid	0.6		0.009
	hexenoic acid	0.2		0.009
	heptenoic acid	0.6		0.01
	octenoic acid	0.2		0.01
	nonenoic acid	1.1		-
Alkanes	pentane	0.4	x	-
	hexane	0.4	x	0.08
	heptane	0.5	x	0.04
	octane	0.5	x	0.03
	nonane	2.5	x	0.1
	decane	0.5		-
Alkenes	butene	x	x	-
	pentene	0.4	x	-
	hexene	0.1	x	0.05
	heptene	0.3	x	0.03
	octene	0.15	x	0.03
	nonene	0.25	x	0.005
Dienes	hexadiene	0.7		0.02
	heptadiene	0.3		0.02
	octadiene	4		0.04
	nonandiene	0.7		-
Aromatics	benzene	0.02	x	0.01
	toluene	0.02	x	-
	ethylbenzene		x	-
	dimethylbenzene		x	-
	trimethylbenzene		x	-

*H_3O^+ ionization mode;
** NO^+ ionization mode
x : detected compound
- : not detected in PTR-ToF-MS spectra
